# Psychometric validation of the Cardiac Distress Inventory - Short Form among people with cardiac diseases in Hong Kong

**DOI:** 10.3389/fpsyt.2024.1412264

**Published:** 2024-12-17

**Authors:** Ted C. T. Fong, Ian C. H. Leung, Chun Ka Wong, Alun C. Jackson, Rainbow Tin Hung Ho

**Affiliations:** ^1^ Centre on Behavioral Health, The University of Hong Kong, Hong Kong SAR, China; ^2^ Research Hub of Population Studies, The University of Hong Kong, Hong Kong SAR, China; ^3^ Department of Medicine, School of Clinical Medicine, The University of Hong Kong, Hong Kong SAR, China; ^4^ Australian Centre for Heart Health, Melbourne, VIC, Australia; ^5^ Department of Social Work & Social Administration, The University of Hong Kong, Hong Kong SAR, China

**Keywords:** Cardiac Distress Inventory, cardiac rehabilitation, Chinese, psychometrics, reliability, resilience, validity

## Abstract

**Objectives:**

Cardiac patients experience various somatic and psychosocial symptoms and stress is an important prognostic factor of cardiac rehabilitation. This study evaluated the psychometric properties of the 12-item Cardiac Distress Inventory – Short Form (CDI-SF) in the Chinese context.

**Methods:**

A total of 227 patients with cardiac diseases were recruited in a specialist outpatient clinic in Hong Kong between Aug 2022 and July 2023. The participants completed the CDI-SF and validated measures on psychosocial functioning and quality of life. Exploratory factor analysis and partial correlation analysis were conducted to examine the factorial validity, reliability, and convergent validity of the CDI-SF with reference to validating measures.

**Results:**

The 1-factor model showed adequate model fit with excellent composite reliability (ω = .92) and substantial factor loadings (λ = .64 –.94, *p* <.01). The CDI-SF factor was negatively associated with age (*r* = –.21, *p* <.01) and showed positive and strong partial correlations (*r* = .59 –.69, *p* <.01) with impact of event, depression, and burnout, and negative partial correlations (*r* = -.43 to -.54, *p* <.01) with resilience and quality of life.

**Conclusion:**

Our study provides the first results on the psychometric properties of the CDI-SF among cardiac patients in Hong Kong. The psychometric results support the CDI-SF as a precise, valid, and reliable measure of cardiac distress in the Chinese context.

## Introduction

Heart diseases such as ischemic heart disease and myocardial infarction are the most common cause of death globally (1) and constituted the third most common cause of mortality in Hong Kong in 2020. Patients with heart disease are prone to somatic and psychological symptoms (2, 3). Stress is increasingly recognized as a prognostic factor among people with cardiac diseases. Cardiac distress is conceptualized as a persistent negative emotional state that challenges the capacity of a patient to cope with the disease, the associated treatments, and the resultant changes to daily living (4). Despite the clinical importance, existing studies in cardiology have mostly assessed the distress of people with cardiac diseases via general measures such as the General Health Questionnaire and Kessler Psychological Distress Scale (5, 6). Assessment of patient-reported outcomes could offer useful insights in cardiovascular clinical practice and improve the quality of care and better inform clinical decisions (7).

Cardiac distress is a complex construct with diverse clusters of symptoms such as fear of death, reduced quality of life, role transition, loss of control, and social isolation (8). A comprehensive assessment tool of cardiac distress would allow clinicians to evaluate the patients’ changes across multiple domains (9) and facilitate better treatment prognosis for the people with cardiac diseases (10). The Cardiac Distress Inventory (CDI) was developed as a new measure of cardiac distress in 405 people with cardiac diseases in Australia (11). The CDI has shown good psychometric properties in terms of validity and reliability for an in-depth, holistic evaluation of cardiac distress. The 55-item CDI has eight subscales: fear and uncertainty, disconnection and hopelessness, changes to roles and relationships, overwhelm and depletion, cognitive challenges, physical challenges, health system challenges, and death concerns. It has been used as a clinical assessment tool within a clinical interview setting.

Although CDI can identify the specific nature of distress in cardiac patients, its length can hinder applications in primary care and cardiac rehabilitation. Recently, the 12-item CDI–Short Form (CDI-SF) was developed by Australian researchers as a screening tool based on the results of Rasch analysis (12), where two of the best items were selected from the four most influential CDI factors with one item chosen from the four remaining factors. The CDI-SF showed a unidimensional factor structure with good reliability, convergent validity, and discriminant validity in Australian patients. Ongoing scale validation studies have translated the CDI-SF from English to various languages such as German, Hebrew, Italian, Arabic, Thai, Turkish, Swedish, Farsi, and French. Importantly, neither the CDI nor the CDI-SF have been validated in the Chinese context. Given the increasing prevalence and risks of cardiovascular disease in China (13), it is important to have a precise and valid measure to enhance our understanding of cardiac distress among cardiac patients and the associated factors in the Chinese context. Resilience refers to the cardiac patients’ ability to cope with and adapt to stressful situations associated with cardiac events (14). Cardiac patients with higher levels of resilience could cope better with the challenges of cardiac diseases and perceive lower levels of distress.

The present study aimed to evaluate the psychometric properties of the Chinese CDI-SF in people with cardiac diseases in Hong Kong: first, to examine the dimensionality, factorial validity, and reliability of the CDI-SF; second, to investigate the convergent validity of the CDI-SF factor with relevant constructs, namely, impact of event, resilience, depression, well-being, and burnout. This study had two hypotheses. In Hypothesis 1, the CDI-SF would show satisfactory factorial validity and reliability. In Hypothesis 2, the CDI-SF would show adequate convergent validity with positive correlations with impact of event, depression, and burnout and negative correlations with resilience and well-being.

## Methods

### Study design and procedures

The present cross-sectional study recruited participants who attended cardiac rehabilitation services in a specialist outpatient clinic of a major public hospital in Hong Kong using convenience sampling. Inclusion criteria included: 1) aged 18 years or above; 2) diagnosis of cardiac disease; 3) ability to understand Chinese; and 4) ability to provide informed consent. Recruitment of patients was promoted via posters and leaflets at the clinic and the clinic staff referred eligible patients to the research team. A trained research assistant approached the potential participants to ascertain their eligibility and intent to join the study. Voluntary participants provided written informed consent and completed a self-report questionnaire while waiting for the appointment with the cardiologist. The questionnaire took 10 minutes to complete. The research assistant would assist the participants in completing the questionnaire and perform basic checks for missing responses.

Data collection took place between August 2022 and July 2023. A total of 227 eligible patients with cardiac diseases voluntarily joined the study (response rate = 25.3%). Study participation was entirely voluntary and the participants could stop the survey at any time without negative consequences. All information provided by the participants was kept strictly confidential. After completing the questionnaire, the participants were provided contacts of emotional support services should they feel distressed. Ethical approval was obtained from the Institutional Review Board of the author’s university (Reference number: UW 22-545).

### Development of the Chinese CDI-SF

The CDI-SF was translated from English into Chinese based on standard guidelines on scale adaptation (15). Permission to translate and use the CDI-SF was obtained from the original developer. The initial translation phase was carried out by two independent translators, who were bilingual Chinese native speakers. One of them was familiar with the measured construct while the other was not. Any discrepancies in the translations were discussed and resolved by consensus. The translated Chinese version was back translated into English by two other bilingual translators, who were unaware of the underlying construct. Afterwards, the research team, consisting of two scholars familiar with the concept of cardiac distress, a cardiologist, and four translators, reviewed all translated versions and resolved any inconsistencies through consensus. This version was finalized through interviews with 10 people with cardiac diseases to ensure the language clarity of the CDI-SF and the preservation of the original meaning.

The CDI-SF comprised 12 items which were answered in a 4-point format (0 = ‘no distress’, 1 = ‘slight distress’, 2 = ‘moderate distress’, 3 = ‘severe distress’) on cardiac distress over the past four weeks (12). Example items of the CDI-SF included “Being physically restricted” and “Having changes in my usual roles”. The total CDI-SF score had a theoretical range from 0 to 36, with a cutoff score ≥ 13 suggested as an indicator of substantial cardiac distress (12). In the present sample, all 12 items displayed positive skewness (1.84 - 6.49) and were treated as ordinal variables in subsequent analyses.

### Measures

The questionnaire assessed demographic information (age, gender, and education level) and clinical characteristics (type of heart disease and duration of diagnosis). The questionnaire pack consisted of five validated measures: Psychological impact of the heart disease was assessed by the 22-item Impact of Events Scale Revised (16). The people with cardiac diseases were instructed to report their degree of life stress as a result of heart disease over the past two months. Three types of psychological responses were measured: intrusive symptoms (8 items), avoidance symptoms (8 items), and hyperarousal symptoms (6 items). The 22 items were rated on a 4-point format from 0 = “not at all” to 3 = “extremely”. Resilience was measured by the 6-item Brief Resilience Scale over the past 4 weeks (14). The items were rated on a 5-point format from 1 = “strongly disagree” to 5 = “strongly agree” and three items were reverse items.

Depressive symptoms were assessed by the 9-item Patient Health Questionnaire (PHQ9) (17). The PHQ9 is a measure of depression over the past two weeks. The items were rated on a 4-point format from 0 = “not at all” to 3 = “almost every day”. Subjective well-being was measured by the 5-item WHO-5 Well-Being Index (WHO-5) (18). The WHO-5 was developed by the World Health Organization and measures the subjective well-being of the patients over the past two weeks. The five items were rated on a 6-point format from 0 = “not at all” to 5 = “always”. Burnout was assessed by the 6-item personal burnout subscale of the Copenhagen Burnout Inventory (19). This scale measured the degree of emotional exhaustion of the respondents over the past two weeks. The items were rated on a 5-point (0-25-50-75-100) format from 0 = “not at all” to 100 = “very often”. All measures showed good to excellent levels of reliability (α = 0.84 – 0.95) in the present sample.

### Data analysis

The CDI-SF items showed substantial floor effects with more than a quarter of the sample endorsing the minimum category. Given the 4-point response scale and asymmetric item distributions, they were treated as ordinal categorical variables in the analysis (20). The present sample minimal missing responses (missing N = 4) in the CDI-SF, which was handled using full information maximum likelihood under the missing-at-random assumption (21). Psychometric properties of the CDI-SF were examined in three steps.

First, the factorial validity of the CDI-SF was evaluated by exploratory factor analysis (EFA) using robust weighted least square estimator (20) with promax rotation in Mplus 8.6 (22). which was adequate for obtaining accurate estimates of validity and reliability in psychometric analysis of the CDI-SF. A 1-factor and 2-factor EFA model for the 12 CDI-SF items had 12 and 23 free parameters, respectively. The present sample (N = 227) had 9.87 – 18.9 cases per parameter, which met the requirement of 10:1 ratios and showed adequate power for obtaining accurate estimates of validity and reliability (23). Dimensionality of the CDI-SF was determined based on model fit, scree plot, and parallel analysis. Problematic items without substantial factor loadings (λ < 0.50) were removed from the model. Model fit was examined based on the following criteria on fit indices: chi-square (χ^2^), comparative fit index (CFI) ≥ 0.95, root-mean-square error of approximation (RMSEA) ≤ 0.06, and standardized root mean square residuals (SRMR) ≤ 0.06 (24).

Second, composite reliability of the CDI-SF factor was evaluated via McDonald Omega (ω) with values ≥ 0.75 indicating good reliability. Third, convergent validity was examined via bivariate correlations between the CDI-SF factor and demographic variables (gender, age, educational level, and duration of diagnosis). The relationships between the CDI-SF factor and validating variables, namely, impact of event, resilience, PHQ9, well-being, and burnout were assessed by partial correlations after controlling for the effects of demographic variables. Cutoff scores on correlations *r* were 0.1, 0.3, and 0.5 for small, medium, and large effect sizes, respectively (25). Statistical significance was set at 0.05 in this study.

## Results

### Sample profile

**Table 1** reports the demographic profile and descriptive statistics of the sample. The mean age of the sample was 59.2 years (SD = 12.8) and the majority of them were males (73.1%). In the sample, the most prevalent type of heart disease was coronary artery disease, followed by arrhythmia. The average duration of heart disease since diagnosis was 7.41 years (SD = 8.91). The sample reported moderate levels of resilience and well-being and low levels of cardiac distress, impact of event, depressive symptoms, and burnout. The 12 CDI-SF items were positively and moderately to strongly correlated (*r* = .44 –.87, *p* <.01) and the mean inter-item correlation was.68 (SD = .10). All 12 items showed significant and substantial item-total correlations (*r* = .52 –.76, *p* <.01).

**Table 1:** Demographic profile and descriptive statistics of the sample (N = 227).

Categorical variables	N (%)	Continuous variables	Range	M (SD)
Gender – Female	61 (26.9)	Age	19 - 87	59.2 (12.8)
Education level:		Diagnosis duration (year)	0 - 54	7.41 (8.91)
Primary	10 (4.4)			
Secondary	99 (43.6)	Impact of Event Scale:		
Bachelor	65 (28.6)	Total impact	0 - 62	12.4 (12.3)
Master or above	53 (23.3)	Intrusion	0 - 23	4.12 (4.80)
Type of heart disease:		Avoidance	0 - 22	5.49 (5.38)
Heart failure	8 (3.5)	Hyperarousal	0 - 17	2.93 (3.54)
Coronary artery disease	78 (34.4)	Resilience	1 - 5	3.41 (0.76)
Unstable angina	8 (3.5)			
Arrhythmia	64 (28.2)	Depressive symptoms	0 - 27	4.56 (4.48)
Acute myocardial infarction	28 (12.3)	WHO-5 well-being	0 - 25	13.2 (6.74)
Cardiac valve disease	25 (11.0)	Burnout	0 - 100	27.9 (22.4)
Cardiac leakage	3 (1.3)			
Others	41 (18.1)	CDI-SF	0 - 36	5.29 (3.06)

### Factorial validity and reliability

**Figure 1:**
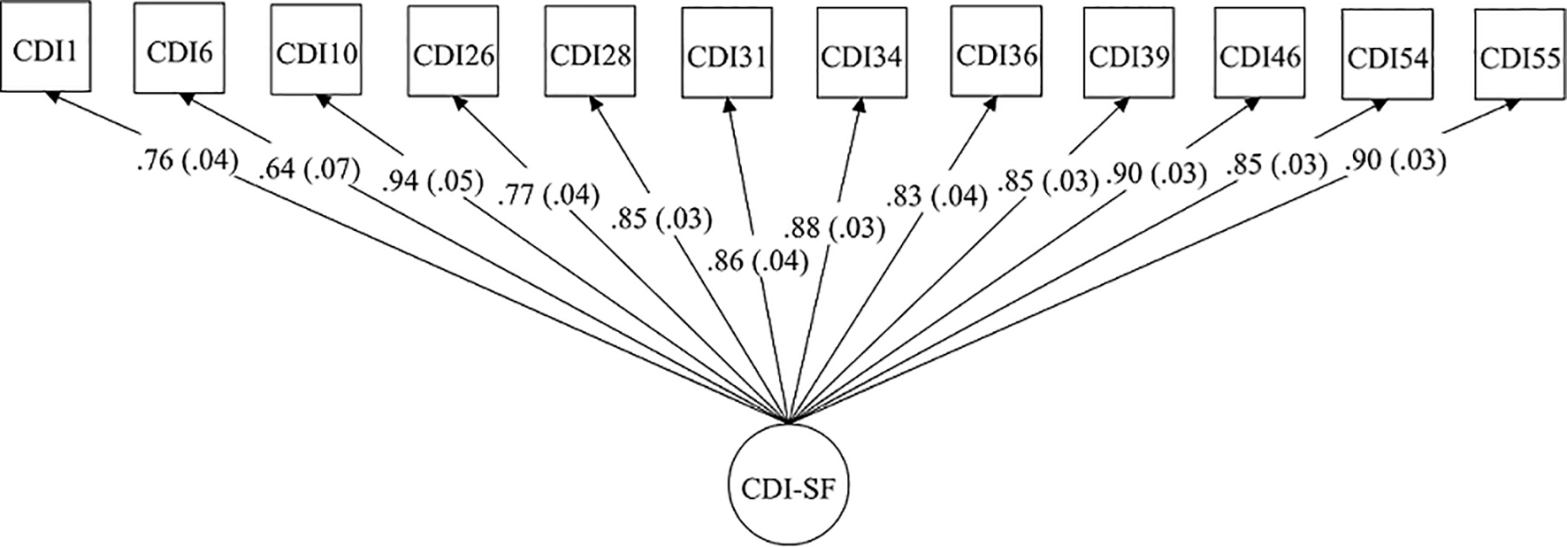
Standardized factor loadings of the 12 CDI-SF items in the 1-factor model. Standard errors are shown in parentheses.

The 1-factor EFA model provided an adequate fit (χ^2^ = 80.5, *df* = 54, *p* = .011, CFI = .99, RMSEA = .047, SRMR = .062) to the data. As **Figure 1** shows, all 12 items showed substantial factor loadings (λ = .64 –.94, *p* <.01) on the CDI-SF factor. Chi-square difference test did not find a superior fit (Δχ^2^ = 19.5, *df* = 11, *p* = .053) for the 2-factor model over the 1-factor model. The EFA model showed eigenvalues of 8.57 for the first factor and 0.70, 0.66, and 0.56 for subsequent factors and the parallel test supported retaining the first factor only. The CDI-SF factor exhibited good composite reliability (ω = .92). 5.8% of the present sample reported a total CDI-SF score ≥ 13.

### Convergent validity

**Table 2** shows the associations between the CDI-SF factor, demographic variables, and validating variables, namely. impact of event, resilience, PHQ9, quality of life, and burnout. The CDI-SF factor was significantly and negatively correlated with age (*r* = –.21, *p* <.01) but not with other demographic variables (*p =* 0.09 – 0.47). After controlling for the effects of demographic variables, the CDI-SF factor showed positive and strong bivariate correlations (*r* = .59 –.69, *p* <.01) with total impact of event, burnout, and depressive symptoms. Moderate to strong correlations (*r* = .44 –.64, *p* <.01) were found between the CDI-SF factor and intrusive, avoidance, and hyperarousal symptoms. Negative and moderate to strong correlations were found between the CDI-SF factor and well-being and resilience (*r* = -.43 to -.54, *p* <.01).

**Table 2:** Associations between the CDI-SF factor, demographic variables, and validating variables on impact of event, resilience, PHQ9, quality of life, and burnout.

	Bivariate correlation
Demographic variables	r (SE)
Gender (Male)	0.06 (0.07)
Age	-0.21 (0.07)**
Education level	-0.05 (0.06)
Duration of heart disease	0.12 (0.07)
	Partial correlation
Validating variables	r_partial_ (SE)
Total impact of event	0.61 (0.04)**
Intrusion	0.56 (0.05)**
Avoidance	0.44 (0.06)**
Hyperarousal	0.64 (0.04)**
Depressive symptoms	0.69 (0.03)**
Burnout	0.59 (0.04)**
Resilience	-0.54 (0.05)**
WHO-5 well-being	-0.43 (0.06)**

N = 227; CDI-SF = Cardiac Distress Inventory - short form; **p <.01. r = bivariate correlations; r_partial_ = partial correlations after controlling for the effects of demographic variables.

## Discussion

The present study was the first to systematically examine the psychometric properties of the CDI-SF in assessing cardiac distress in cardiac patients in Hong Kong. Our EFA results supported the unidimensional structure of the CDI-SF as a valid and parsimonious latent structure of cardiac distress in the Chinese context. Overall, the present study found consistent results with the scale development study in Australia (12). The strong factorial validity and reliability of the CDI-SF provided empirical support for Hypothesis 1. Despite a lack of significant gender difference, younger patients in the present sample showed significantly higher levels of cardiac distress. Our results support adequate convergent validity of the CDI-SF and Hypothesis 2 with substantial partial correlations with validating measures in the expected directions. Patients with higher levels of cardiac distress reported greater impacts of their cardiac event, higher depressive symptoms and burnout, and worse well-being. A recent study found similar associations between mental distress and burnout and well-being in community adults under the COVID-19 pandemic (26).

Resilience denotes the coping ability of cardiac patients to cardiac disease and has been shown to be protective against cardiovascular disease risks (27, 28). Consistent with previous findings (29), the present study found a substantial and negative association between resilience and cardiac distress. This implies that patients who were more resilient would cope better with cardiac disease and perceive lower levels of cardiac distress. A meta-analysis found that more resilience resources at multiple levels were associated with better cardiovascular outcomes in American samples (30). Longitudinal studies are needed to elucidate the temporal protective effects of resilience on subsequent cardiovascular health and overall quality of life via cardiac distress. A 3-wave panel study has found significant and beneficial indirect effects of social support and hope on quality of life via emotional distress in stroke survivors (31). Future research could examine whether the CDI-SF plays a potential mediating role in the temporal effects of impact of cardiac event on psychosocial outcomes of the cardiac patients. These findings could elucidate the underlying neurobiological and psycho-behavioral mechanisms in cardiac rehabilitation programs for amelioration of the cardiac distress symptoms (9, 32).

The present findings support the CDI-SF as a brief, valid, and reliable measure of cardiac distress in the Chinese context. Using the recommended CDI-SF cutoff score of ≥ 13, 5.8% of the present sample was classified to have clinical levels of cardiac distress, which was comparatively lower than the prevalence (28.9%) observed in the Australian study (12). Our present sample of cardiac patients had average illness duration of 7.4 years while the Australian study mainly comprised cardiac patients having an acute coronary event in the last 12 months. It was plausible that the chronic illness nature of our sample contributed to the difference in the prevalence of cardiac distress. The present study provided the first results on the psychometric properties of the CDI-SF in the Chinese context outside the original development study in Australia (12). Results from ongoing validation studies of the CDI-SF in other languages such as German, Italian, Turkish, and Thai are needed to enrich our understanding of cardiac distress across cultural contexts through international comparative studies.

From a clinical perspective, the CDI-SF could be a useful screening tool in regular clinical checkups to identify patients with elevated risks of distress related to cardiac disease. This not only enables early identification of cardiac patients with greater vulnerabilities, but also facilitates better treatment planning and monitoring of the treatment progress. Subsequent administration of the 55-item original CDI would provide a comprehensive assessment of distress symptoms (12), which would enable provision of holistic and person-centered care to cardiac patients according to their service needs in corresponding priority areas. Resilience-building interventions such as cognitive behavioral therapy and positive psychology interventions could promote the coping skills and self-efficacy of cardiac patients. These could, in turn, lead to better symptom management and coping with stress in their rehabilitation process.

## Limitations

There are a few study limitations. First, the present sample was recruited via convenience sampling in a single specialist outpatient clinic. The non-random sampling design and low response rate (25.3%) have implications for the sample representativeness. Given potential non-response bias, caution is warranted in generalizing the present results to a broader population of Chinese people with cardiac diseases. Second, the cross-sectional design and modest sample size (N = 227) of the present study did not permit testing of measurement invariance across time and demographic subgroups. Longitudinal studies with larger sample sizes are suggested to evaluate the measurement invariance of the CDI-SF across time, gender, age groups, and types of heart disease. Third, the present study did not investigate the discriminant validity of the CDI-SF with regard to clinical diagnosis. Future studies should conduct receiver operating characteristic curve analysis to compare the diagnostic ability of the CDI-SF in detecting substantial psychological distress to generic distress scales such as the Chinese Health Questionnaire (33) in the Chinese context. Fourth, the present study did not include measures on other relevant constructs such as personality, cardiac self-efficacy, and sleep disturbance (34). Previous studies have highlighted the clinical relevance of sleep disorders in people with cardiac diseases, and further studies are needed to elucidate the linkages between sleep disturbance and cardiac distress symptoms. Fifth, the present study focused on the relationships among the study variables at the aggregate level. Future research could utilize the network approach to investigate the comorbidity among sub-domains of cardiac distress at a symptom level for fine-grained results (35).

## Conclusions

This study evaluated the psychometric properties of the CDI-SF as a brief measure of cardiac distress in the Chinese context. Overall, the scale demonstrated satisfactory levels of factorial validity, reliability, and convergent validity with impact of cardiac events, depression, burnout, resilience, and quality of life of the patients. The results support the use of CDI-SF as a precise, valid, and reliable measure of assessing cardiac distress in clinical and research settings. Future studies should examine the clinical utility of CDI-SF in long-term prognosis and care planning for patients with cardiac diseases.

## Data Availability

The raw data supporting the conclusions of this article will be made available by the authors, without undue reservation.
